# Real-Time Estimation and Monitoring of COVID-19 Aerosol Transmission Risk in Office Buildings

**DOI:** 10.3390/s23052459

**Published:** 2023-02-23

**Authors:** Jelle Vanhaeverbeke, Emiel Deprost, Pieter Bonte, Matthias Strobbe, Jelle Nelis, Bruno Volckaert, Femke Ongenae, Steven Verstockt, Sofie Van Hoecke

**Affiliations:** IDLab, Ghent University-imec, 9052 Ghent, Belgium

**Keywords:** COVID-19 aerosol transmission risk estimation, dynamic dashboard, streaming architecture, indoor sensors, IoT, semantic data, building management

## Abstract

A healthy and safe indoor environment is an important part of containing the coronavirus disease 2019 (COVID-19) pandemic. Therefore, this work presents a real-time Internet of things (IoT) software architecture to automatically calculate and visualize a COVID-19 aerosol transmission risk estimation. This risk estimation is based on indoor climate sensor data, such as carbon dioxide (CO_2_) and temperature, which is fed into Streaming MASSIF, a semantic stream processing platform, to perform the computations. The results are visualized on a dynamic dashboard that automatically suggests appropriate visualizations based on the semantics of the data. To evaluate the complete architecture, the indoor climate during the student examination periods of January 2020 (pre-COVID) and January 2021 (mid-COVID) was analyzed. When compared to each other, we observe that the COVID-19 measures in 2021 resulted in a safer indoor environment.

## 1. Introduction

Because of the coronavirus disease 2019 (COVID-19) pandemic, countries have required employees to work from home and imposed strict safety measures. Now, employees are allowed to partially or completely return to the office and may have to follow certain guidelines, such as increasing ventilation or limiting occupancy, to help reduce the indoor transmission of COVID-19 [1].

Monitoring the indoor carbon dioxide (CO_2_) concentration has become a widespread preventive strategy. Multiple governments have suggested keeping the CO_2_ level below, for example, 900 ppm [2,3]. Should this threshold be exceeded, action must be taken to increase ventilation or reduce occupancy. While monitoring this threshold is straightforward in practice with the help of CO_2_ sensors, it is too generic to be true in every situation. Researchers have addressed this issue and proposed more advanced models that estimates a safe CO_2_ concentration based on the indoor and epidemiological situation [4,5]. However, this research remains largely unknown and unused by the general public since commercially available CO_2_ monitors do not provide such a functionality. These only display the measured CO_2_ on a screen, and at best show a color coding according to some fixed thresholds.

Furthermore, while these CO_2_ devices are very convenient to use, they only show the results locally. This becomes especially problematic for large buildings where building managers require a good overview of every room for which an Internet of things (IoT) system is needed to collect and store the data centrally. The application of IoT systems during the COVID-19 pandemic has also been discussed for many other domains, ranging from personal health tracking to contact tracing [6,7]. Next to IoT data collection, software tools need to be provided to make monitoring and analysis as easy as possible for users and building managers. This is often performed through mobile or web applications that visualize the data in a dashboard. Many real-time air quality monitoring software architectures were presented in the past that include all necessary components to monitor a complete building [8,9,10]. However, in order to implement more advanced COVID-19 risk estimation models, a powerful and flexible data stream processing platform is needed, which is missing in existing works. Furthermore, dashboards are often tailor-made for one use case, but users cannot easily adapt them to other applications or needs.

The purpose of our work is two-fold. Firstly, these recent COVID-19 aerosol transmission risk estimation models are applied into practice so occupants can benefit from their research. Secondly, a complete software architecture that collects, processes and visualizes the sensor data stream is proposed.

To achieve this first goal, two existing models [5,11] were combined and translated into a methodology for the real-time estimation of the COVID-19 aerosol transmission risk. Since the focus is on office buildings, some assumptions can be made on, for example, the activity level of the occupants, helping make the model more practical without being too general. This methodology is then also converted into a declarative SPARQL query which can be executed by the second part of the research, i.e., the real-time monitoring software architecture.

The proposed software architecture is built to be scalable and flexible in every aspect. First, the sensor data are ingested and stored centrally. Subsequently, the stream processing is performed by microservices that first semantify the data, after which semantic technologies, i.e., SPARQL, can be used to query the data stream and link it with other static semantic building data. As a result, these stream processing services are not only limited to the use case of this paper, but can handle a large range of other applications, such as general air quality monitoring and comfort scoring. Lastly, the visualization is performed by a dynamic dashboard that suggests visualizations based on the semantics of the data. Additionally, the dynamic dashboard is also capable of receiving events, e.g., a high-COVID-19 transmission risk, and notify the users of this.

This work will allow occupants and building managers to monitor the estimated COVID-19 aerosol transmission risk so that they can take action when needed. Furthermore, it will give the tools to implement and monitor a large variety of other building management applications.

### 1.1. Main Contributions

The main contributions of this work are:

- Combination of existing models into a methodology for COVID-19 aerosol transmission risk estimation;
- Creation of a flexible and scalable software architecture to collect, process and visualize large data streams;
- Implementation of a real-time stream processing microservice to execute the defined COVID-19 aerosol transmission risk estimation on incoming semantic sensor data linked with static semantic building data;
- Visualization in a dynamic dashboard which suggests appropriate visualizations based on the semantics of the selected data;
- Automatic notification of occupants and building managers when the estimated COVID-19 risk is too high.

### 1.2. Structure of the Research Paper

The next section will briefly discuss the existing related work concerning COVID-19 risk estimation and mitigation, and real-time monitoring systems. Some of this literature is then used to establish the risk estimation methodology discussed in Section 3. Afterwards, Section 4 will go over all elements in our software architecture, from sensors to visualization, which make it possible to employ the methodology in large buildings. Section 5 then discusses the strengths and weaknesses of the proposed work. In Section 6, the research is applied in a practical setting in order to compare the impact of on-campus COVID-19 measures. Finally, the conclusions and future research directions are discussed in Section 7.

## 2. Related Work

### 2.1. COVID-19 Safety

Some models that estimate the risk of COVID-19 aerosol transmission, the probability of infection and/or safe CO_2_ concentration already exist. Peng et al. [4,12] proposed a model and spreadsheet (https://tinyurl.com/covid-estimator) (accessed on 25 July 2022) with multiple examples for common indoor environments, such as a classroom, supermarket or stadium. The parameters can also be adapted for other custom room and viral conditions. Similarly, Bazant et al. [5,13] created a model and online web application (https://indoor-covid-safety.herokuapp.com) (accessed on 27 July 2022) that uses parameters such as room dimensions, human activity, etc., to derive the maximum allowed exposure time or safe CO_2_ concentration for an occupant to that room before exceeding a specific transmission risk. Both tools provide dynamic guidelines depending on the indoor environment but lack the ability to provide real-time feedback.

On the other hand, Pang et al. [14] trained an artificial neural network based on the results of computational fluid dynamics (CFD) simulations to predict the COVID-19 infection risk with respect to the CO_2_ concentration. A smart ventilation control system was developed using this model in order to reduce the infection risk in the building. While this system dynamically optimizes the indoor environment, it does not provide feedback to the occupants for them to take action.

Others have worked on tools to provide the real-time validation of COVID-19 safety and check whether certain guidelines have been respected. Petrović and Kocić [15] created an IoT architecture that checks social distancing and the wearing of masks with a camera-based system, while also using semantic technologies to link all data. Numerous other literature for social distance validation is available, for example [16,17]. However, these works do not investigate the risk of aerosol transmission.

### 2.2. Real-Time Monitoring Systems

Many real-time monitoring software architectures exist with different focuses on, for example, indoor air quality, indoor comfort and energy consumption. The literature study by Saini et al. reviewed 40 air quality monitoring systems, finding answers to questions such as the used sensors and wireless technologies [18]. Below, some interesting and closely related works are briefly discussed.

Marques et al. proposed the iAirCO_2_ system for which they developed their own air quality sensor based on the Arduino hardware platform [8]. As the name of their system suggests, the main focus lies on CO_2_ analysis. The captured CO_2_ data are transmitted using a Wi-Fi connection to their own software architecture. Users can access and analyze the data via a web and smartphone application which are built using open source technologies. Next to that, they also give the option to set CO_2_ thresholds, which produce a notification when exceeded. Although the system is advertised as modular, there is currently no possibility of easily adding additional microservices to perform more advanced processing on the data stream.

Similarly, Benammar et al. proposed a modular IoT platform for real-time indoor air quality monitoring [9]. Instead of focusing on just CO_2_, they added a range of additional air quality sensors that measure, for example, sulfur dioxide (SO_2_) and nitrogen dioxide (NO_2_). This gives a more complete view of the indoor air quality than Marques et al. [8]. Furthermore, the sensor network is designed differently. Instead of directly transmitting the data through Wi-Fi, the sensors are part of a Zigbee mesh network and send their data to a gateway which allows the sensors to be more energy-efficient. The gateway then takes care of the data transmission over Wi-Fi to the webserver. One of the main focuses of this work was ensuring a reliable communication between the sensors, gateway and webserver. Hence, a data logging and retry mechanism is present on the gateway in case of an Internet outage. For data processing and visualization, the open source Emoncms platform was used, which allows for a lot of flexibility but does not take advantage of semantic technologies to, for example, allow the linking of the sensor data to other sources.

Another interesting work is the SAMBA system proposed by Parkinson et al. [10]. It consists of a neat and comprehensive hardware device that monitors the indoor environmental parameters at a desk space and transmits its data to the gateway using a Zigbee mesh network topology. The gateway itself is equipped with cellular technologies, e.g., long-term evolution (LTE), to transmit the data to a centralized web service called IEQAnalytics. There, the data are processed, effectively checking the compliance of the environment with different indoor environment quality standards. An online dashboard visualizes the measured parameters and environment quality indices. The complete SAMBA system is an accurate and user-friendly means of monitoring the indoor environment quality. However, the visualizations in the dashboard are predefined and cannot be adapted by users with different needs.

### 2.3. Conclusions

Previous research has focused on creating models to estimate the aerosol transmission risk of a certain environment, controlling the building’s ventilation system, creating IoT systems that assess social distance or mask wearing, or building software systems for indoor environmental monitoring. However, to the best of our knowledge, no work has presented a flexible and scalable software architecture that uses semantic technologies to estimate and visualize the COVID-19 aerosol transmission risk based on real-time sensor measurements in office buildings.

## 3. COVID-19 Aerosol Transmission Risk Estimation

The COVID-19 risk estimation methodology provides an automatic scoring mechanism of the indoor office environment conditions. Although this estimation does not represent the real virological risk, it is a valuable tool to easily assess whether an indoor climate is favorable for limiting the transmission of COVID-19. Not only does this help increase the awareness of employees regarding the impact of the indoor climate on COVID-19 transmission but it also allows them to take action if the estimated risk is high.

This methodology combines the proposed work of two literature sources. The first and main part of this risk estimation relies on the work of Bazant et al. [5] who proposed a model to estimate safe CO_2_ concentration in a room. To further take the thermal properties of the room into account, the research of Spena et al. [11] was applied to estimate the viral load survival which is then fed into the model of Bazant et al. to improve its estimation.

**Figure 1:**
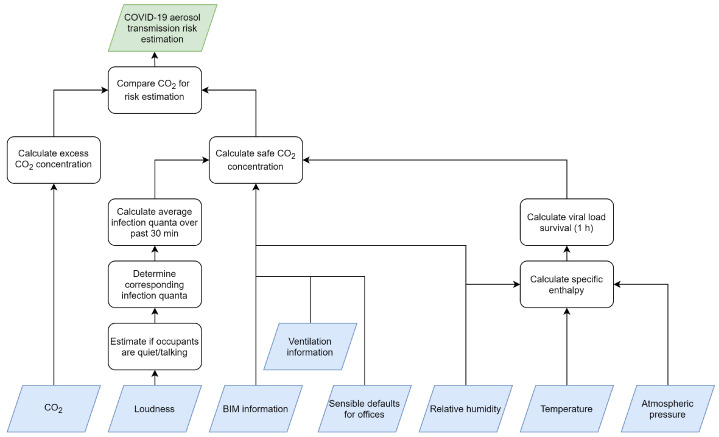
Overview of the coronavirus disease 2019 (COVID-19) aerosol transmission risk estimation methodology.

Figure 1 gives an overview of the complete methodology with all its inputs (sensors, building information model (BIM), etc.) at the bottom and the resulting risk estimation at the top. The executed calculation steps for safe CO_2_ concentration, viral load survival and risk estimation will be discussed in more detail below.

### 3.1. Safe CO_2_ Concentration

Researchers agree that aerosols are one of the main ways of COVID-19 transmission [19,20]. Whereas respiratory droplets caused by coughing and sneezing are one form of airborne transmission, aerosols are small virus particles produced by exhalation that can float longer in the air than respiratory droplets. This results in a risk of transmission to people in the same room, even when no close contact took place. Therefore, proper ventilation has become one of the key means of reducing the risk of indoor COVID-19 transmission [1].

Because of the importance of ventilation, this risk estimation methodology validates the air quality of a room based on CO_2_ measurements. Although the CO_2_ concentration is not a perfect reflection of ventilation and air quality, it is often used as a proxy [4,5,21] as it can be measured using CO_2_ sensors which are cheap, widely available and easily retrofittable in existing buildings. However, this is only valid when people are the main source of CO_2_ in a room. Then, increasing CO_2_ values indicate that stale air builds up quicker than the room is ventilated. This poses a risk when an infected person is in the same room since higher CO_2_ levels correlate with more rebreathed air and thus a higher chance of inhaling virus particles.

The safe CO_2_ concentration of the risk estimator is dynamically determined based on the work of Bazant et al. [5] who proposed a model to calculate this for a given environment. The calculated safe CO_2_ threshold can then easily be compared to real CO_2_ sensor readings. We refer to the paper of Bazant et al. [5] for the details of their model; however, in the following paragraphs, a discussion of a few important parameters and how they could be configured in order to apply the model in a practical environment is conducted. Most of the suggested default values were obtained from the accompanying web application (https://indoor-covid-safety.herokuapp.com) (accessed on 27 July 2022).

A first and important parameter is the prevalence of COVID-19, specified by the percentage of the population which is infected. It is best to determine this based on the effective local COVID-19 infection statistics, but in case these are not available, some literature states that a fixed prevalence value of 0.1% is a possible base case to work with [4]. Equally, for the immunity percentage, it is possible to look at the real local immunity and vaccination rates, but it can also be set to, for example, zero for a more conservative guideline. Every now and then, a new variant emerges which is more infective than the previous. Therefore, it is also important to take the current dominant viral strain into account. Currently, the omicron strain is the most widespread [22], to which this parameter should be set accordingly. The risk tolerance is a parameter that can be freely chosen based on the use case and the desired safety level. The web application suggests a default of 10%.

Another important parameter is the exposure time in the room. The longer occupants are in a room, the longer they are exposed to the potential risk of transmission. The duration of a working day varies from country to country, but according to 2021 statistics, the average working week ranged from 32 to 40 h in Europe [23], resulting in an average workday of approximately 8 h. We use this value as the exposure time in the model of Bazant et al. [5].

The model also requires information on the size of the room. Since a lot of modern office buildings, including ours, have a BIM model available, that can be used to extract the size information per room. Moreover, the ventilation of a room is also of high importance. If some of this information, such as the ventilation rate, is available in the building management system (BMS), it can be directly used from there. Otherwise, the ventilation control scheme and technical installation files can provide the information needed to make a good estimation of these parameters, i.e., the ventilation rate, filtration and recirculation. The relative humidity in the room can be provided in real-time by installed environmental sensors.

Not only are the properties of the pandemic and room included, but the filtration efficiency of facial masks is also considered. This parameter could be dynamically set based on mask detection using a computer vision system, but an easier and more privacy-friendly way would be to define it according to the corporate mask policy. For example, if masks are not enforced, the filtration efficiency can be set to zero.

How much CO_2_ and virus aerosols are produced is influenced by the occupant’s activity. Since this methodology applies the work of Bazant et al. [5], specifically in an office environment, it is assumed that the occupants will be mostly sitting and not performing heavy physical activities. On the other hand, whether the occupants are talking varies. Therefore, a coarse-grained differentiation is made between two activities, i.e., breathing and talking, based on decibel level sensor data, as shown in Figure 1. When the measured decibel level is lower or equal than 50 dB, we assume that everyone is working quietly in the room and that the production of CO_2_ and virus particles is at the rate of breathing. A decibel level higher than 50 dB could indicate that some of the occupants are talking which allows adjusting of the model’s parameters accordingly. The 50 dB threshold was empirically determined based on the historical sensor data of our offices but can be chosen differently for other environments. This elementary estimation has its limitations (e.g., sound intensity decreases with distance, other sources of noise can be present, etc.), but the simplicity and fact that it is non-privacy intrusive are a clear advantage.

Table 1 shows the examples of the predicted safe CO_2_ concentration by the model of Bazant et al. [5] for the two different activities, breathing and talking. These calculations were performed using the web application, leaving most of the parameters at their default, to show the significantly different result between the two activities. As can be seen, the safe excess CO_2_ concentration for talking is approximately 4 times smaller than for breathing, which is due to the infection quanta of talking being much larger. Note that these are just examples, and the effective safe CO_2_ concentration will be dynamically calculated by the system described in Section 4 based on the real parameters of the room.

The large difference in the safe CO_2_ threshold of breathing and talking would also lead to large fluctuations in the risk estimation if occupants go from one activity to the other. This could confuse the occupants and ultimately demotivate them to further monitor it. Therefore, not only could the current decibel measurement be used to decide which infection quanta to apply, but all decibel readings of the past 30 min were used to calculate the average infection quanta for that period, as shown in Equation (1). This allows for a more smooth transition of the quanta, and thus risk estimation, between these two activities.

(1) $$q_{avg}=\frac{n_{breath}*q_{breath}+n_{talk}*q_{talk}}{n_{breath}+n_{talk}}$$

where:

$$\begin{array}{cc} \;q_{avg} & =\mathrm{the}\;\mathrm{average}\;\mathrm{infection}\;\mathrm{quanta}\;\left(q/m^{3}\right)\\ n_{breath} & =\mathrm{the}\;\mathrm{number}\;\mathrm{of}\;\mathrm{decibel}\;\mathrm{measurements}\;\mathrm{considered}\;\mathrm{breathing}\;(\leq 50\mathrm{dB});\\ n_{speak} & =\mathrm{the}\;\mathrm{number}\;\mathrm{of}\;\mathrm{decibel}\;\mathrm{measurements}\;\mathrm{considered}\;\mathrm{speaking}\;(>50\mathrm{dB});\\ q_{breath} & =\mathrm{the}\;\mathrm{infection}\;\mathrm{quanta}\;\mathrm{for}\;\mathrm{breathing}\;(q/m^{3});\\ q_{talk} & =\mathrm{the}\;\mathrm{infection}\;\mathrm{quanta}\;\mathrm{for}\;\mathrm{speaking}\;(q/m^{3}). \end{array}$$

### 3.2. Viral Load Survival

The thermal conditions of the environment also have an influence on the risk of transmission. While these are integrated in the model of Bazant et al. [5] through the effect of the “viral decay rate” parameter, other literature more extensively estimate this, incorporating both the temperature and humidity conditions. Although research is not consistent and shows a different relation and significance, the common trend is that COVID-19 spreads more in a cold and dry indoor climate [11,25]. Therefore, it is recommended to optimize the indoor temperature and humidity in buildings to reduce the survival time of COVID-19 aerosols. An additional benefit of a higher humidity is that aerosol droplets will grow due to hygroscopy and fall down quicker. This reduces the time that virus particles are airborne and thus reduces their ability to travel long distances [26].

For a more extensive estimation of the viral survival time, the COVID-19 research performed by Spena et al. [11] was incorporated. Temperature, relative humidity and atmospheric pressure were used to calculate the specific enthalpy, which is a measure of the energy in a thermodynamical system. Based on the literature data of COVID-19 and other related viruses, Spena et al. studied the relation between the specific enthalpy and the viral load survival after 1 h ($VLS_{1h}$) for different thermal conditions, which revealed a quadratic relation, as shown by Equation (2). This quadratic relation is used by our methodology to estimate the viral load survival based on the real-time indoor conditions, and is then converted into the viral decay rate in order to pass it to the model of Bazant et al. [5].

(2) $$VLS_{1h}=\left\{\begin{array}{cc} 1 & \mathrm{if}\;h\leq 38\;\mathrm{or}\;h\geq 67\\ C_{1}h^{2}+C_{2}h+C_{3} & \mathrm{otherwise} \end{array}\right.$$

where

$$\begin{array}{cc} \;VLS_{1h} & =\mathrm{viral}\;\mathrm{load}\;\mathrm{survival}\;\mathrm{after}\;1\;h\;\left(\%\right)\\ h & =\mathrm{specific}\;\mathrm{enthalpy}\;(kJ/kg_{dry\;air})\\ C_{1} & =0.0047562426\\ C_{2} & =-0.4994054697\\ C_{3} & =13.1093935791 \end{array}$$

### 3.3. Risk Estimation

With the safe CO_2_ concentration calculated based on the room’s conditions, it is possible to take the last step, namely estimating the COVID-19 aerosol transmission risk. Therefore, the current excess CO_2_ concentration needs to be calculated based on the last real-time CO_2_ measurement. There are multiple options to tackle this. The easiest but incomplete option is to use the global average CO_2_ level, which is currently about 415 ppm [24]. A second and better option is to find the minimum sensor reading over a period of time (e.g., 1 week) and use the found minimum as the background CO_2_ level. This assumes that the room will be completely ventilated at least once during the chosen period. Once the background CO_2_ level is known, the excess CO_2_ concentration can be easily calculated by subtracting the background CO_2_ from the measured CO_2_ concentration. Afterwards, the measured excess CO_2_ can be compared to the estimated safe excess CO_2_ concentration by taking the ratio between both values as shown in Equation (3). This results in a unitless number specifying the estimated risk. As a final step, this number is clipped between 0 and 1 which gives us the final COVID-19 aerosol transmission risk estimation.

(3) $$RE=CO_{2,measured}/CO_{2,safe}$$

where

$$\begin{array}{cc} \;RE & =\mathrm{risk}\;\mathrm{estimation}\\ CO_{2,measured} & =\mathrm{the}\;\mathrm{measured}\;\mathrm{excess}\;{\mathrm{CO}}_{2}\mathrm{concentration}\;(\mathrm{ppm})\\ CO_{2,safe} & =\mathrm{the}\;\mathrm{safe}\;\mathrm{excess}\;{\mathrm{CO}}_{2}\mathrm{concentration}\;(\mathrm{ppm}) \end{array}$$

## 4. Real-Time Monitoring System

**Figure 2:**
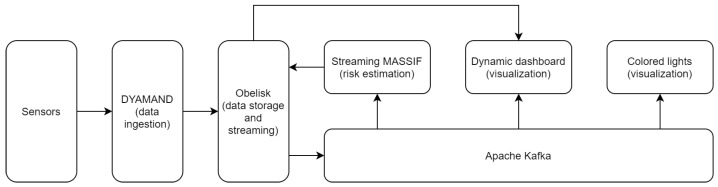
Overview of the microservice architecture.

The real-time monitoring of our COVID-19 aerosol transmission risk is handled by a modern software architecture which is a combination of microservices fulfilling different tasks, such as data ingestion, persistence, risk estimation, and visualization in a dashboard. Using a microservice architecture provides fault isolation, eliminates long-term commitment to a single technology stack and is easily extendable by adding services later on. These microservices can also be easily scaled up or down to handle different load scenarios. The overview of the microservice architecture is given in Figure 2, of which the individual components will be detailed in the following subsections.

### 4.1. Sensors

Slightly over a hundred Netatmo Smart Home Weather stations (https://www.netatmo.com/en-row/weather/weatherstation) (accessed on 28 January 2022) were installed to measure the environmental conditions in every room of our office building. These are off-the-shelf commercially available sensors that measure the environmental parameters for the COVID-19 aerosol transmission risk estimation presented in Section 3. These sensors communicate through Wi-Fi which makes the integration in existing buildings easy as those usually already have a Wi-Fi infrastructure. The Netatmo stations are placed at desk height and in the center of the room, away from ventilation exhausts or inlets and windows, which can have a significant impact on the measurements. The nondispersive infrared (NDIR) CO_2_ sensor auto-calibrates itself once a week by setting the minimum measured value to 400 ppm. Every 10 min, the environmental parameters are measured and transmitted.

Nevertheless, our monitoring system is not limited to a specific type or brand of sensor. It can process environmental data from any source since the DYAMAND [27] middleware is used in the system architecture which serves as an interoperability layer that abstracts and standardizes the communication between smart devices and applications. This standardization ensures that no hardware-specific adaptations need to be made further down the processing pipeline.

### 4.2. Semantic Sensor Metadata

All sensors are accompanied by metadata which are stored as a semantic graph. For this, the standard semantic sensor network (SSN) ontology [28] is used which is specifically designed for the description of sensor networks and their measurements. These metadata allow us to easily query the data, for example, selecting all sensors that measure CO_2_. Additionally, this also facilitates the linking of different parts of information, such as the location, which is, on its turn, also semantically described. Each room in the building is available in the ontology with the information extracted from the BIM model, such as the room’s name, floor, area, etc.

**Figure 3:**
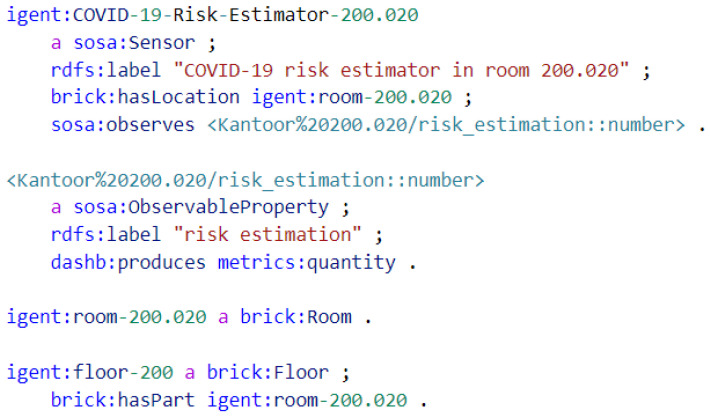
Semantic annotation in Turtle format of a COVID-19 risk estimator, describing it as a (virtual) sensor with a location and the COVID-19 aerosol transmission risk estimation as observed property.

Similarly to how the physical sensors and their measurements are described semantically, the COVID-19 risk estimator is also defined as an, albeit virtual, semantic sensor, as shown in Figure 3. By doing this, the results are linked to the room and available for further usage just like any sensor.

### 4.3. Ingestion, Persistence and Messaging

As mentioned in Section 4.1, the Netatmo sensors transmit their measurements through Wi-Fi to the DYAMAND middleware which standardizes the data. Afterwards, the result is transmitted to Obelisk [29], a scalable IoT integration platform, which both stores the data and streams them onto Apache Kafka (https://kafka.apache.org) (accessed on 16 March 2022). As shown in Figure 2, this Kafka bus is the central element in our architecture and allows for the easy addition of new services while still decoupling them all.

### 4.4. Streaming MASSIF

**Figure 4:**
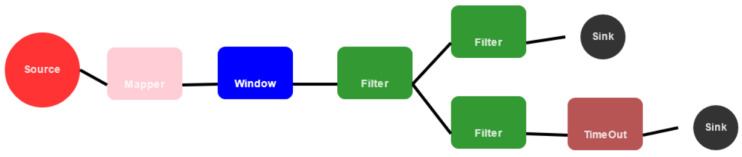
Streaming MASSIF pipeline to calculate the COVID-19 aerosol transmission risk estimation.

With the sensor data streamed onto the Kafka bus, different applications can now make use of it. In our software architecture, Streaming MASSIF [30] is used to process these sensor data. Streaming MASSIF is an in-house designed stream processing platform that solves data velocity and variety by exploiting stream reasoning techniques. Targeting velocity is necessary for our scenario as the sensors continuously produce data. Variety needs to be targeted as the sensor observations need to be combined with static data, such as the semantic sensor ontologies. The Streaming MASSIF web interface [31] is used to set up the complete processing pipeline of the COVID-19 aerosol transmission risk estimation, as shown in Figure 4. An overview of the components will be given in the following paragraph.

The first component, left in Figure 4, is a “source” which is the point where the sensor data come in. Multiple source types are available, but here the source was configured to read all data of a certain topic from the Kafka bus. When data come in, these are sent to the next component which is a “mapper”. As the name implies, this component can map the incoming messages to another format. In this case, it is used to transform the sensor data from Kafka into a semantic structure which allows us to use semantic technologies, i.e., SPARQL, in the later steps. Then, the data are passed to the “window” component. This collects and groups all data over a given time period (window size) and outputs them with a certain interval (stride). For this use case, the window size is set to 30 min which allows us to calculate statistics and perform aggregations over a longer period of time. The stride is set to 10 min since the sensors also transmit their data at this rate. With each stride, the window data are passed to the next block which is a “filter”. This versatile and powerful component allows us to write any SPARQL query to act on the semantic messages. Next to that, ontologies can also be included in order to link additional semantic data, such as the sensor descriptions and locations. This filter block provides the implementation and execution of our COVID-19 aerosol transmission risk estimation using a declarative SPARQL query which consists of three major steps: firstly, all required data are queried, secondly, the calculations are performed, and lastly, the results are outputted semantically. After this filter, the pipeline branches into two (see Figure 4): one branch for feeding the calculated risk estimations back into the software architecture (upper); and one to throw events when the risk estimation in a room is too high (lower). The upper branch consists of another “filter”, containing a simple SPARQL query to select the risk estimations of the previous block, and a “sink” that sends the output to the configured destination. Just like the “source” component, multiple destinations are available, but Obelisk is chosen in this case. The lower branch also starts with another “filter” that selects the risk estimations and checks whether they are higher than a set threshold. If that is the case, an event graph is constructed. The following “time out” block makes sure that the event is not sent to the “sink” more than once in a certain period, e.g., 1 h.

### 4.5. Dynamic Dashboard

The last step in the COVID-19 risk monitoring architecture was the dynamic dashboard where users can monitor the building status and be alerted of events and anomalies. Dashboards need to balance between flexibility and ease of use. Classical dashboards require the user to specify and configure the widgets of each desired visualization. To solve this shortcoming, our in-house designed semantic dashboard [32] dynamically suggests suitable visualizations by reasoning over the sensors’ semantic descriptions and supported visual widgets.

**Figure 5:**
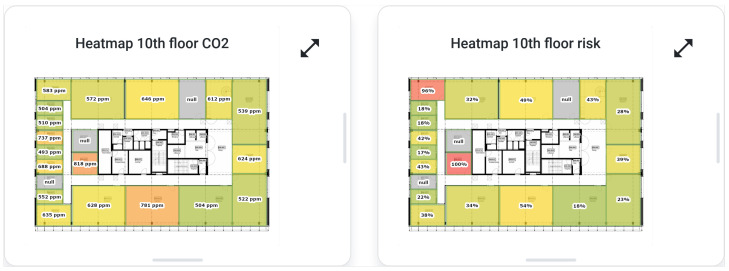
Heatmap visualization of the CO_2_ concentration and COVID-19 aerosol transmission risk estimation of the 10th floor of our office building.

For the use case at hand, a new visualization was added to dynamically visualize the sensor values (e.g., CO_2_, COVID-19 risk estimation, etc.) as a heatmap. The visualization is based on floor plans acquired from the BIM model of the building. Every room is colored based on the requested sensor value which gives an easy overview of the different rooms. Figure 5 depicts such floor plans, visualizing the CO_2_ concentration or risk estimation.

#### 4.5.1. Visualization Suggestion

**Figure 6:**
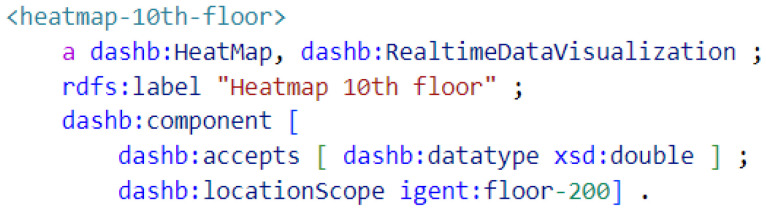
Semantic description in Turtle format of the real-time heatmap visualization for the 10th floor of our office building.

The dashboard dynamically suggests visualizations based on the selected sensor(s). This suggestion is made by semantic reasoning over the metadata of sensors and visualizations. Reasoning allows our approach to be loosely coupled, facilitating the reuse of the same functionality on sensors described with another ontology. The visualizations are annotated using our own dashboard ontology. Figure 6 shows the annotation of a heatmap visualization. The most important fields in the semantic metadata are “dashb:accepts” which describes the supported datatypes, and “dashb:locationScope” which describes the location that this visualization is relevant for. Thus, in the example of Figure 6, one can see that the heatmap accepts any quantitative data (xsd:double) and is only relevant for sensors located on the 10th floor. When the user selects one or multiple sensors, the reasoning engine will infer which visualizations are compatible by ensuring that the sensor data type and location match those accepted by the visualization.

#### 4.5.2. Event View

The dynamic dashboard is not only capable of visualizing data upon request by the user, but it can also react to events. Whenever the COVID-19 risk estimation exceeds a defined threshold, Streaming MASSIF sends out an event to Obelisk which is then again pushed to Kafka (see Figure 2). As the dashboard is listening to the Kafka, it automatically and dynamically constructs a new tab based on this event containing information about the signal that triggered the event, a textual description, the occurrence time, and optionally multiple stimuli. A stimulus is a data signal that influenced the event and is added to the event view tab as a visualization widget. The dashboard reasoner is used on all data signals to dynamically infer which visualization is best suited.

**Figure 7:**
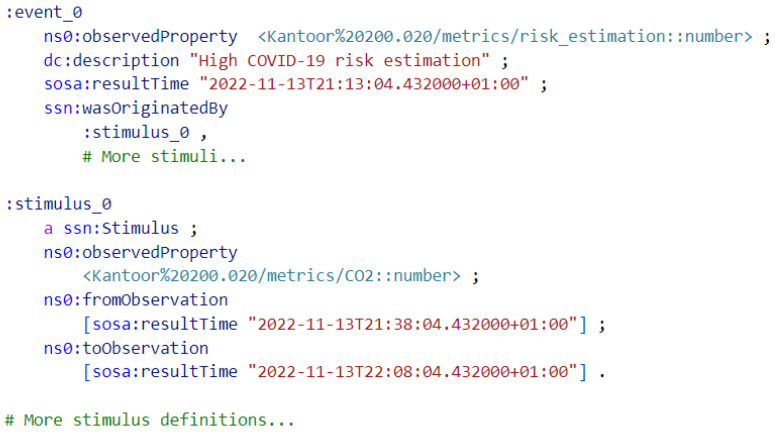
Semantic annotation in Turtle format of a “High COVID-19 risk estimation” event produced by Streaming MASSIF. The event is linked to the COVID-19 aerosol transmission risk estimation metric of a room, the occurrence time and multiple stimuli.

**Figure 8:**
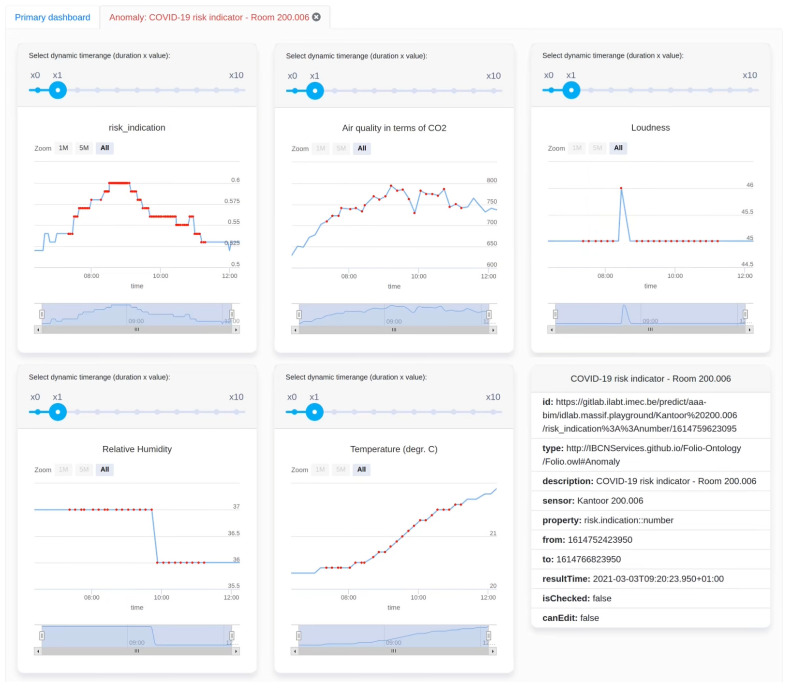
Dynamically constructed event view when a “High COVID-19 risk estimation” event occurs, visualizing the COVID-19 aerosol transmission risk estimation signal and linked stimuli.

Figure 7 describes an example of a “High-COVID-19 risk estimation” event produced by Streaming MASSIF. In this case, the stimuli are the sensor measurements used to calculate the risk estimation. An example of the resulting dynamically created dashboard tab is shown in Figure 8.

### 4.6. Colored Lights

**Figure 9:**
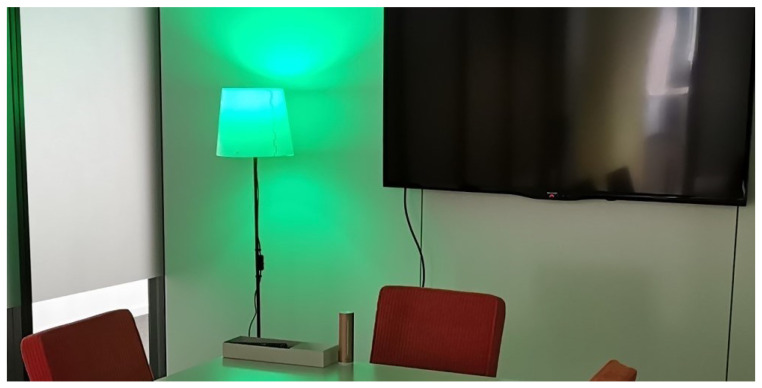
A colored light showing the real-time COVID-19 aerosol transmission risk estimation status in a meeting room.

While the dashboard is available for all employees, probably not everyone will check it continuously during the day. Next to that, our offices are often visited by external partners and students who do not have access to the dashboard. Therefore, a second and more direct visualization is provided by means of colored lights. Smart Yeelight (https://en.yeelight.com) (accessed on 10 August 2022) light bulbs were installed in free-standing lamps in every office and meeting room, as can be seen in Figure 9. The lights are controlled by a microservice that listens to the Kafka bus (see Figure 2). Three possible colors are set according to the risk estimation: green when the risk estimation is ≤0.6, orange when it is >0.6 and <0.9, and red when it is ≥0.9.

## 5. Strengths and Weaknesses

### 5.1. COVID-19 Aerosol Risk Estimation

The risk estimation methodology heavily relies on CO_2_ data which has both benefits and limitations. Its main advantage is the ease of use since CO_2_ is easily measured using sensors while still being a good proxy for indoor air quality [4,5,21]. This comes with the important assumption, and possible limitation, that people must be the main source of CO_2_ in the room. Only then will CO_2_ levels correlate with the respiratory activity of occupants in the room. When other sources are present, e.g., gas furnaces, this assumption is no longer true, and the analysis of CO_2_ data will give misleading results. Another possible weakness is the importance of the placement of the CO_2_ sensors. On the one hand, these need to be located close to the used desk space, but on the other hand, they should not be too close to persons, windows, doors, and ventilation.

Besides CO_2_, our risk estimation methodology also takes advantage of other environmental parameters, such as temperature and humidity. These are equally practical to measure since they are often incorporated in one measurement device. By using these additional sensor values, our methodology can give a more complete view of the COVID-19 aerosol transmission risk than checking a fixed CO_2_ threshold. On the downside, the methodology also needs a lot of additional parameters on the building. Some are easy to set according to preference or policy, but others can be more difficult to provide and may require the help of a building manager.

The estimation of the occupant’s activity is another strength of this methodology. While the approach for this is basic, it still brings an important improvement since breathing and speaking have vastly different infection quanta. Nevertheless, it can also be incorrectly influenced by, for example, loud background noise in which case a more advanced sound analysis would be beneficial.

Lastly, this methodology only focuses on giving information to occupants and building managers. The responsibility of acting when needed is up to them. Depending on the use case, it might be more interesting to control the ventilation system directly as in [14].

### 5.2. Real-Time Monitoring System

The use of commercial environmental sensors can be seen as a benefit and limitation at the same time. On the one hand, these sensors can be bought in large numbers and are guaranteed to work if from a trustworthy vendor. On the other hand, the available functionality is provided by a third party and can sometimes be a limiting factor. Related works building their own sensor device are more flexible in that respect.

The complete architecture is designed to be performant, flexible and modular. Obviously, this comes with some complexity as well. This type of system is not meant to be set up by a non-technical user, such as a CO_2_ monitor, but requires experience in the deployment and maintenance of the Kubernetes cluster containing all the components. Another downside is the dependence on Internet access of our system. Currently, there is no fallback mechanism in the case of an Internet outage as in the research by [9].

The Kafka bus in our software system enables a microservice architecture that brings a multitude of benefits, such as flexibility, scalability, fault isolation, etc. Different services can easily plug into the bus to bring additional functionalities. One of the services in our case is Streaming MASSIF, which allows us to use semantic technologies to process the sensor data stream. This brings the benefit that all data are semantically described and linked, from observation to room, allowing the implementation of a large variety of use cases besides this COVID-19 risk estimation. A downside is that knowledge of semantic technologies is required.

Similar strengths are true for our dynamic dashboard, which uses the power of semantic data to achieve a flexible and user-friendly interface. The users can add visualizations according to their preference and receive suggestions for these based on the selected data. The dashboard also receives events and automatically visualizes all included information, which helps in finding and analyzing problems and risks in the building.

## 6. Functional Evaluation: Impact of On-Campus COVID-19 Measures

The COVID-19 risk estimation was evaluated by comparing the statistics of two auditoriums of our university building during a pre- and mid-COVID-19 period. As the pre-COVID-19 period, the examination session of January 2020 was used. COVID-19 was not present in Belgium back then, so exams were made on-campus without any increased ventilation or occupancy restriction. As the mid-COVID-19 period, the examination session of the following year, January 2021, was used. Then, COVID-19 was a worldwide pandemic and restrictions applied in Belgium. On-campus exams were only allowed with a limited number of students and increased ventilation was required. Comparing the risk estimations of these two periods allows us to see whether the enforced guidelines and rules effectively resulted in a safer indoor environment.

Normally, for the calculation of the risk estimation, the prevalence of COVID-19 should be dynamically set based on the local epidemiological statistics, but since none are available for January 2020 (pre-COVID-19), that would lead to unusable results for comparison. Therefore, the prevalence was set to a fixed value of 0.1% for both examination periods to allow us to compare the effect of the additional guidelines on the risk estimations. Both examination periods were equally long and thus have an equal amount of data samples to process.

**Figure 10:**
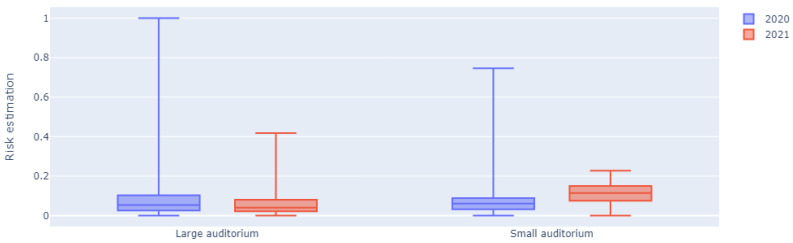
COVID-19 aerosol transmission risk estimation box plots of the examination periods of January 2020 and January 2021 for two auditoriums.

Figure 10 shows the box plots of the risk estimations of a large and small auditorium during the examination period of January 2020 and 2021. For the large auditorium, it is clear that the maximum risk estimation is much lower in 2021 than 2020 thanks to the applied guidelines and restrictions. Additionally, the 25% quantile, 75% quantile, and mean risk estimations are also lower in 2021. For the small auditorium, the maximum risk estimation is also much lower in 2021 compared to 2020, again showing the positive impact of the safety rules. However, the other statistics are higher because this small auditorium was used more frequently in 2021. Students had to be spread across multiple rooms to give everyone the opportunity to take the exam at the same time, resulting in higher room occupation than normal.

To statistically test whether the risk estimations in 2021 were lower than in 2020, an alternative hypothesis was defined accordingly and the *p*-values were calculated based on the data of the large and small auditoriums. Since the data were not normally distributed, the Mann–Whitney U test was used. As shown in Table 2, the *p*-value for the large auditorium was far below p<0.05, and is thus statistically significant, suggesting that our alternative hypothesis is true and that the taken COVID-19 measures had a positive impact on the estimated COVID-19 aerosol transmission risk. The *p*-value for the small auditorium on the other hand is far above the significance level of α=0.05, thus rejecting the alternative hypothesis. This is in line with our expectation, given the fact that this small auditorium was more frequently used in 2021 as mentioned earlier.

## 7. Conclusions and Future Research

Given the importance of aerosol transmission of COVID-19, we designed and implemented a real-time software architecture to estimate and monitor the COVID-19 transmission risk in office buildings, helping to increase the awareness of occupants and safety inside. The platform is able to ingest large volumes of environmental sensor data in real time with the help of Obelisk. Then, the Kafka bus enables a versatile and scalable microservice architecture, allowing processing and visualization services to be easily added. The COVID-19 aerosol transmission risk estimation was implemented as an intuitive pipeline in Streaming MASSIF which lets us combine the power of semantic and stream processing technologies. The resulting risk estimations can be visualized on the floor plan widget of our dynamic dashboard to enable occupants and building managers to grasp the COVID-19 risk estimations with one glance. Finally, the colored lights bring an even more accessible visualization inside our building. The proposed software system is not only limited to the COVID-19 aerosol transmission risk estimation, but is flexible and modular, allowing the addition of other building management use cases, such as general indoor air quality testing and comfort scoring. Next to the practical deployment of this system in our university building, a comparison of the estimated COVID-19 aerosol transmission risk was performed in two auditoriums during a pre- and a mid-COVID-19 examination period. The estimated risk in January 2021 for the large auditorium was statistically significantly lower than in January 2020, showing the positive impact of the COVID-19 safety measures taken in 2021. The same conclusion could not be drawn for the small auditorium. However, this small auditorium was also used more often during the examination period of January 2021 since students had to be spread across rooms.

Multiple aspects of this work can still be improved in future research. Currently, the position of the installed sensors is only known at the room level. To make our risk estimation more spatially fine-grained, the location in the room should be further refined by linking the exact sensor position to the BIM model. Next to that, the elementary activity estimation based on decibel-level sensor measurements could be improved by analyzing the complete sound signal instead. This would increase the hardware and processing demands of our system, but should allow for a better and more fine-grained estimation of the different speaking levels, i.e., whispering, normal talking, loud talking, etc. Currently, making the floor plan views is a manual task that needs to be performed once when setting up a new building. Since the BIM model is already available, that will be used in future work to automatically generate the floor plan visualizations and link the sensors to their respective locations on the map. Another step that could be taken in future research is the implementation of edge processing capabilities. Now, all the processing for the COVID-19 risk estimation is executed in the cloud by Streaming MASSIF, but large parts of the computations can be offloaded to an edge device, benefitting the scalability of Streaming MASSIF. Finally, COVID-19 research is still heavily ongoing, so the methodology of the risk estimation should be updated to reflect future insights into the virus and its transmission.

## Figures and Tables

**Table 1:** Example of safe carbon dioxide (CO_2_) concentrations for the occupant activities—breathing (working quietly) and talking.

	Breathing (≤50 dB)	Talking (>50 dB)
Prevalence	0.001	0.001
Risk tolerance	0.1	0.1
Viral strain	Omicron BA.2	Omicron BA.2
Exposure time	8 h	8 h
Floor area	200 m²	200 m²
Respiratory activity	Breathing (4.2 q/m³)	Talking (72 q/m³)
Safe excess CO_2_	850 ppm	209 ppm
+ background CO_2_ [24]	1265 ppm	624 ppm

**Table 2:** *p*-values calculated with the Mann–Whitney U test checking whether the COVID-19 aerosol transmission risk estimations in 2021 are lower than in 2020 for the large and small auditorium.

	*p*-Value
Large auditorium	1.13 × $10^{-16}$
Small auditorium	1

## Abbreviations

The following abbreviations are used in this manuscript:

BIM	Building information model
BMS	Building management system
CFD	Computational fluid dynamics
COVID-19	Coronavirus disease 2019
CO_2_	Carbon dioxide
IoT	Internet of things
LTE	Long-term evolution
NDIR	Nondispersive infrared
NO_2_	Nitrogen dioxide
SSN	Semantic sensor network
SO_2_	Sulfur dioxide
